# ROS in cancer therapy: the bright side of the moon

**DOI:** 10.1038/s12276-020-0384-2

**Published:** 2020-02-14

**Authors:** Bruno Perillo, Marzia Di Donato, Antonio Pezone, Erika Di Zazzo, Pia Giovannelli, Giovanni Galasso, Gabriella Castoria, Antimo Migliaccio

**Affiliations:** 1 Istituto di Scienze dell’Alimentazione, C.N.R., 83100 Avellino, Italy; 2 Istituto per l’Endocrinologia e l’Oncologia Sperimentale, C.N.R., 80131 Naples, Italy; 3 Dipartimento di Medicina di Precisione, Università della Campania “L. Vanvitelli”, 80138 Naples, Italy; 4 Dipartimento di Medicina Molecolare e Biotecnologie Mediche, Università di Napoli “Federico II”, 80131 Naples, Italy

**Keywords:** Health sciences, Cancer therapy, Therapeutics

## Abstract

Reactive oxygen species (ROS) constitute a group of highly reactive molecules that have evolved as regulators of important signaling pathways. It is now well accepted that moderate levels of ROS are required for several cellular functions, including gene expression. The production of ROS is elevated in tumor cells as a consequence of increased metabolic rate, gene mutation and relative hypoxia, and excess ROS are quenched by increased antioxidant enzymatic and nonenzymatic pathways in the same cells. Moderate increases of ROS contribute to several pathologic conditions, among which are tumor promotion and progression, as they are involved in different signaling pathways and induce DNA mutation. However, ROS are also able to trigger programmed cell death (PCD). Our review will emphasize the molecular mechanisms useful for the development of therapeutic strategies that are based on modulating ROS levels to treat cancer. Specifically, we will report on the growing data that highlight the role of ROS generated by different metabolic pathways as Trojan horses to eliminate cancer cells.

## Cancer: A Trojan horse to kill cancer cells

Highly reactive molecules called reactive oxygen species (ROS), which at low levels are natural regulators of important signaling pathways in cells, might be recruited to act as “Trojan horses” to kill cancer cells. Researchers in Italy led by Bruno Perillo of the Institute of Food Sciences in Avelllino review the growing evidence suggesting that stimulating production of natural ROS species could become useful in treating cancer. Although ROS production is elevated in cancer cells it can also promote a natural process called programmed cell death. This normally regulates cell turnover, but could be selectively activated to target diseased cells. The authors discuss molecular mechanisms underlying the potential anti-cancer activity of various ROS-producing strategies, including drugs and light-stimulated therapies. They expect modifying the production of ROS to have potential for developing new treatments.

## Introduction

Reactive oxygen species (ROS) produced in eukaryotic cells through aerobic metabolism have evolved as regulators of important signaling pathways. ROS, previously considered mere byproducts of cellular respiration, are oxygen-containing molecules with high reactivity. They include hydroxyl (HO^*^) and superoxide (O_2_^*^) free radicals and nonradical molecules, such as hydrogen peroxide (H_2_O_2_), which is less reactive than the majority of ROS but is able to reach any cellular compartment prior to being converted by peroxiredoxins and glutathione peroxidases into water and oxygen. In fact, H_2_O_2_ plays the role of a second messenger in some pathways that involve the transduction of extracellular signals and the control of gene expression, contributing to what is currently defined as redox signaling^1^.

ROS are produced in mitochondria (mainly *via* the electron transport chain, where ~1–2% of O_2_ is reduced to form superoxide anions), peroxisomes (through the β-oxidation of fatty acids) and the endoplasmic reticulum (through the oxidation of proteins). Oxidative phosphorylation in mitochondria involves four electron-transporting complexes and a proton-translocating ATP synthase that direct electrons derived from the initial oxidation of NADPH and FADH2 along a multistep pathway that culminates in protons being pumped outside of mitochondria. ROS are also continuously generated by enzymatic reactions involving cyclooxygenases, NADPH oxidases (NOXs), xanthine oxidases and lipoxygenases and through the iron-catalyzed Fenton reaction; indeed, it should be noted that NOXs have primarily evolved to produce ROS^2^. Finally, ROS are generated after exposure to physical agents (ultraviolet rays and heat) and after chemotherapy and radiotherapy in cancer.

Tight regulation of ROS levels is crucial for cellular life; in fact, moderate ROS contribute to the control of cell proliferation and differentiation. Therefore, eukaryotic cells benefit from a complex scavenging system based on superoxide dismutases (SODs), located in the cytoplasm, mitochondria and the extracellular matrix; glutathione peroxidase (GPX); glutathione reductase (GR); peroxiredoxin; thioredoxin; and catalase, which convert superoxide anions into water and recycle the antioxidants in the reduced state.

Here, we focus on the molecular mechanisms that support the elaboration of anticancer therapies that modulate the production and scavenging of ROS and, in particular, on the opportunities raised by their ability to induce cell death upon exceeding a threshold level.

## Biological outcomes of oxidation by ROS

It has been determined that each cell is exposed to ~1.5 × 10^5^ oxidative hits per day. If, for any reason, ROS production increases or the number of scavenged ROS decreases, then cells experience a condition known as oxidative stress. Oxidative stress has been implicated in the pathophysiology of cancer: in fact, high levels of ROS generated by ongoing aerobic glycolysis followed by pyruvate oxidation in mitochondria (the Warburg effect), increase receptor and oncogene activity, and the stimulation of growth factor-dependent pathways or oxidizing enzymes induce genetic instability^3,4^. Moreover, excessive intracellular levels of ROS may damage lipids, proteins and DNA, and this ability has been exploited in a series of anticancer strategies, as detailed below.

### ROS and lipids

By interacting with lipids, ROS can induce oxidative stress through a feedback loop initiated by the peroxidation of fatty acids, which alters the lipid bilayer of cell membranes and generates free radicals. This process is potentially dangerous to cells, as peroxidation of mitochondrial phospholipids may affect the integrity of permeability transition pores (PTPs) and disaggregate complexes I and III of the respiratory chain, thereby enhancing electron leakage within the mitochondrial intermembrane space^5,6^. However, free radicals produced by lipid peroxidation are short-lived^7^.

### ROS and cytoplasmic signaling

By interacting with proteins, ROS have an impact on several signaling pathways involved in the control of cell proliferation and apoptosis. The underlying mechanism generally consists of the oxidation of redox-reacting cysteine and/or tyrosine residues located within or near the active site, which creates intraprotein and interprotein bridges that affect protein function^8,9^. These modifications are reversible and generate a wide array of cellular responses^10^.

In general, phosphatases are inhibited by ROS^11^, whereas kinases may be inhibited or activated^12^. In particular, ROS activate nonreceptor protein kinases belonging to the Src family; small G proteins, such as Ras; and the tyrosine kinase receptors of growth factors^13,14^, as well as components of the c-Jun N-terminal kinase (JNK) and p38 kinase (p38MAPK) pathways that induce apoptosis^15^. Specifically, through the formation of disulfide bonds between catalytic cysteines, H_2_O_2_ inactivates phosphatase and tensin homolog phosphatase (PTEN) and unlocks the phosphoinositide 3-kinase (PI3-K)-dependent recruitment of its downstream kinases, such as protein kinase B (Akt)^16^, or oxidizes the redox protein thioredoxin and thus suppresses its inhibitory effect on the p38MAPK signaling cascade^17^. Intuitively, small increases in ROS would be expected to activate the PI3-K/Akt pathway preferentially, while further increases would be expected to trigger p38MAPK-dependent apoptosis.

ROS also influence the activity of calcium channels; in fact, they induce the release of calcium from cellular stores with the consequent activation of kinases, such as protein kinase C (PKC), thereby playing important roles in the proliferation of cancer cells^18^.

### ROS and nuclear signaling

Most ROS-sensitive pathways transduce cytoplasmic signals to the nucleus, where they influence the activity of transcription factors that control the expression of a wide array of genes. In this regard, to prevent excessive intracellular ROS, cancer cells respond to oxidative stress by inducing the transcription of antioxidant enzymes, highlighting the relevance of an in-depth knowledge of these pathways for use in elaborating therapies that alter ROS levels.

The pivotal redox-sensitive transcription factor is nuclear factor erythroid 2-related factor 2 (Nrf2)^19^, recognized as the leading transcription factor driving the antioxidant response in cancer cells. Under normal conditions, Nrf2 is degraded through its interaction with Kelch-like ECH-associated protein 1 (Keap1), whereas under oxidative stress conditions, Keap1 is oxidized and Nrf2 is translocated to the nucleus, where it induces the expression of several genes^19^. Nrf2 controls the production of glutathione (GSH), the leading antioxidant molecule within cells, through the expression of the enzyme that catalyzes the rate-limiting reaction of GSH synthesis, glutamate-cysteine ligase (GCL), and GSH utilization and regeneration^20,21^. It also controls free Fe(II) homeostasis, upregulating the expression of heme oxygenase HMOX1, which generates free Fe(II) *via* the breakdown of heme molecules. Since Fe(II) catalyzes the Fenton reaction to produce the free radical OH^*^ from hydrogen peroxide, its upregulation presents a paradox: Nrf2 also boosts the expression of the genes encoding several components of the ferritin complex that detoxifies Fe(II) by converting it to Fe(III) and then stores it^22^. Notably, high serum concentrations of ferritin have been described in several cancers with a poor prognosis^23^.

The forkhead box O (FOXO) family of transcription factors is activated by JNK after ROS levels are increased and induces the expression of SODs and catalase^24^. The activation of SODs by a FOXO transcription factor (FOXO4) appears to contradict their antioxidant effect; however, the hydrogen peroxide generated by SODs from O^*^ is the substrate of catalase^25^.

Another important transcription factor that plays a major role in the control of antioxidant gene expression is p53. In fact, the role of p53 in the control of ROS levels is controversial, as it may promote both oxidant and antioxidant gene expression. Indeed, moderately elevated ROS levels inhibit p53, while higher levels promote its expression. Among the targets of p53 activity are sestrins (sestrin 1 and 2) that induce the activity of peroxiredoxins, increasing the impact of the cellular antioxidant array^26^. In this way, p53 has a complementary function to that of FOXO transcription factors that induce the expression of sestrin 3^27^. Interestingly, both p53 and FOXO control a distinct set of genes that are not targets of Nrf2 activity, even though all three factors induce HMOX1 expression and, therefore, Fe(II) storage and secretion, that plays a role in breast tumorigenesis, highlighting the role of antioxidants in cancer promotion^28^.

It is known that a widespread characteristic of tumors is their inability to develop adequate blood vessels with the consequence being relative hypoxia: at moderate levels, ROS induce transcription of HIF1α, the founding member of the family of hypoxia-induced factors, and stabilize the encoded protein, which is normally hydroxylated within less than 5 min, by inhibiting the activity of the iron-dependent prolyl 4-hydroxylase (PHD) involved in its degradation^29^. As a consequence of HIF1α activation, several genes important for cancer progression, such as VEGF and VEGF receptors, are induced^30^.

Finally, the DNA-binding ability of some transcription factors is directly influenced by ROS. For example, ROS, *via* oxidation of thioredoxin, enhance the nuclear localization of both the ataxia-telangiectasia mutated (ATM) serine/threonine kinase, which is involved in DNA damage repair^31,32^, and redox factor-1 (Ref-1), a multifunctional protein that enables Fos/Jun DNA-binding and is identical to the apurinic/apyrimidinic 1 (APE1) endonuclease^33^. The latter factor is able to interact with thioredoxin to reduce a specific cysteine (Cys-62) in the Rel-homology domain (RHD) of the NF-kB subunit p50 that had been previously oxidized by ROS^34^, restoring its ability to interact with specific responsive DNA sequences^35,36^. These data show that ROS can either activate or suppress the NF-kB signaling involved in the control of several important cellular processes, such as embryogenesis and cell proliferation and death, and the responses to a variety of stress stimuli^37^.

### ROS and chromatin

ROS influence the activity of epigenetic modulators, such as histone deacetylases (HDACs) or DNA methyltransferases (DNMTs) with consequences that are evident in the expression of the target genes^38,39^. They also oxidize DNA, especially adenine and guanine (8-oxo-A and 8-oxo-G). It has been reported that ~1 in 10^5^ guanines is oxidized in normal cells and that this proportion is increased by 35–50% in transformed cells^7^. Unrepaired 8-oxo-G is potentially one of the most mutagenic lesions, since it pairs with A, inducing G → T transversions^40^, and represents a prominent candidate to be a marker of ROS-induced mutagenesis and tumorigenesis^41^. Oxidized Gs also impact the methylation of DNA, as indicated by reports showing that damaged bases on the DNA nascent strand can suppress the methylation of a cytosine within a distance of one or two base pairs^42^. However, ROS are able to induce DNA hypermethylation as well, with potential consequences on tumor phenotype when promoter regions of tumor suppressor genes are involved^43–45^. In addition, 8-oxo-Gs accumulate at telomeres, where they inhibit telomerase and decrease the binding of telomeric proteins, leading to the disruption of telomere length and precluding the maintenance of chromosomal-end capping^46^.

Finally, ROS also induce mutations in mitochondrial DNA with the potential generation of a feedback loop in which mutations in genes encoding complexes of the ETC may directly affect the efficiency of electron transport. The major sensitivity of mitochondrial DNA to ROS-induced mutagenesis is intuitive, as this DNA is not protected by histones, and mitochondria lack the nucleotide excision repair (NER) enzymatic system.

**Fig. 1:**
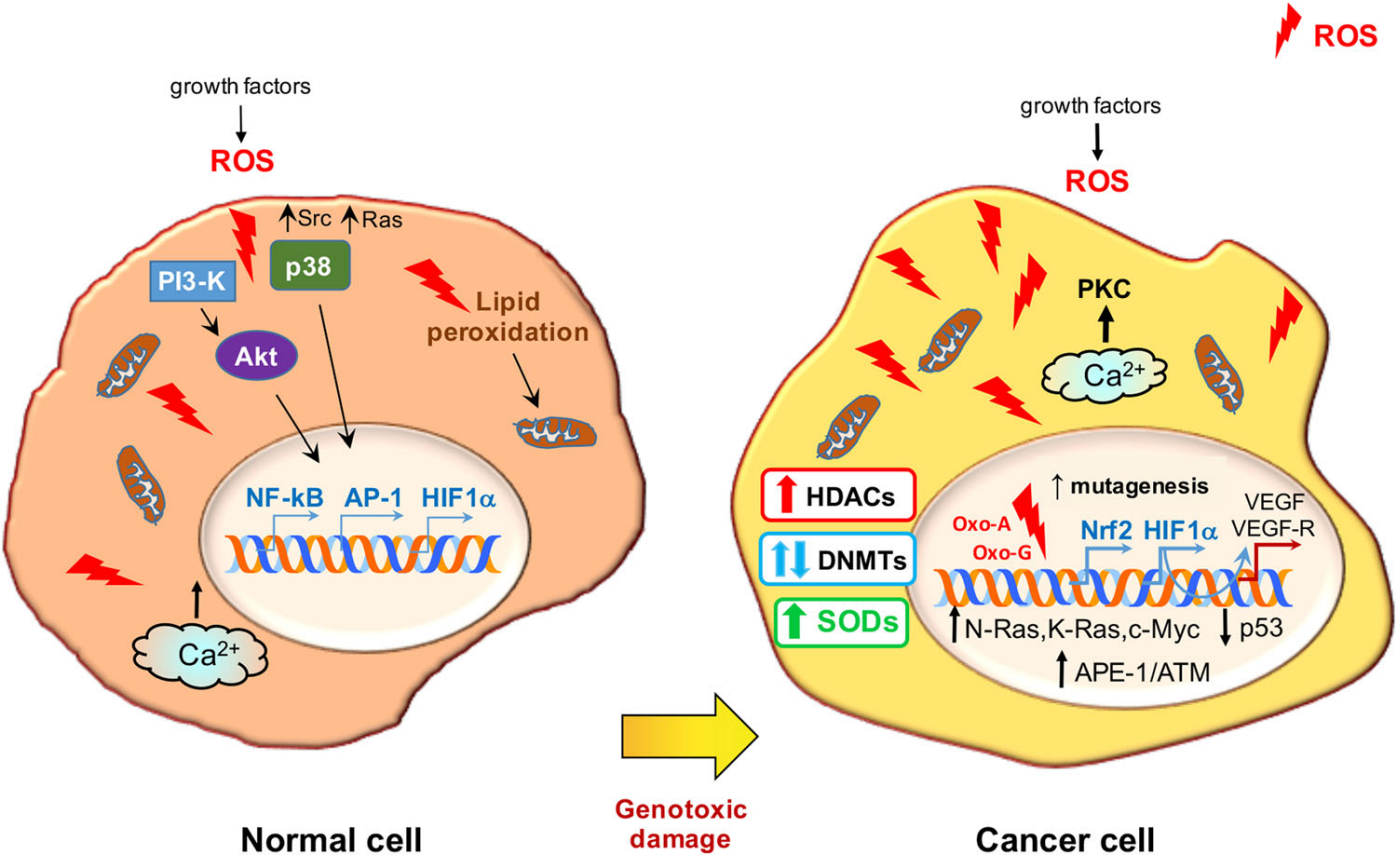
Redox signaling and oxidative stress in normal and cancer cells. The major signaling cascades induced by growth factor-stimulated ROS are highlighted on the left. The same pathways influence the cell cycle and affect the activity of transcription factors and genes that play roles in the cellular response to the hypoxic microenvironment. ROS also induce lipid peroxidation with commensurate electron leakage in mitochondria and the release of Ca^2+^ from intracellular stores. The main consequences of oxidative stress in cancer cells are illustrated on the right. Moderately elevated ROS induce oncogenes and inhibit tumor suppressor genes that, in turn, increase ROS levels. Ca^2+^ release induces PKC, while the expression of genes involved in the formation of new blood vessels and in the establishment of a boosted antioxidant system is enhanced. ROS also activate HDACs and have a dual effect on DNMTs with important outcomes for the expression of oncogenes and tumor suppressor genes. Oxidized bases trigger mutations and engage DNA repair enzymes.

The main consequences of redox signaling and oxidative stress in normal and cancer cells are presented in Fig. 1.

## Oxidative stress promotes cancer and reveals its Achilles heel

Cancer is the second cause of death worldwide and is characterized by several hallmarks^47^; cell transformation, genome instability, hyperproliferation, immortalization, angiogenesis, epithelial-mesenchymal transition (EMT) and metastasis, which are all influenced in several ways by intracellular ROS^48,49^.

### ROS as double-edged swords in cancer

**Fig. 2:**
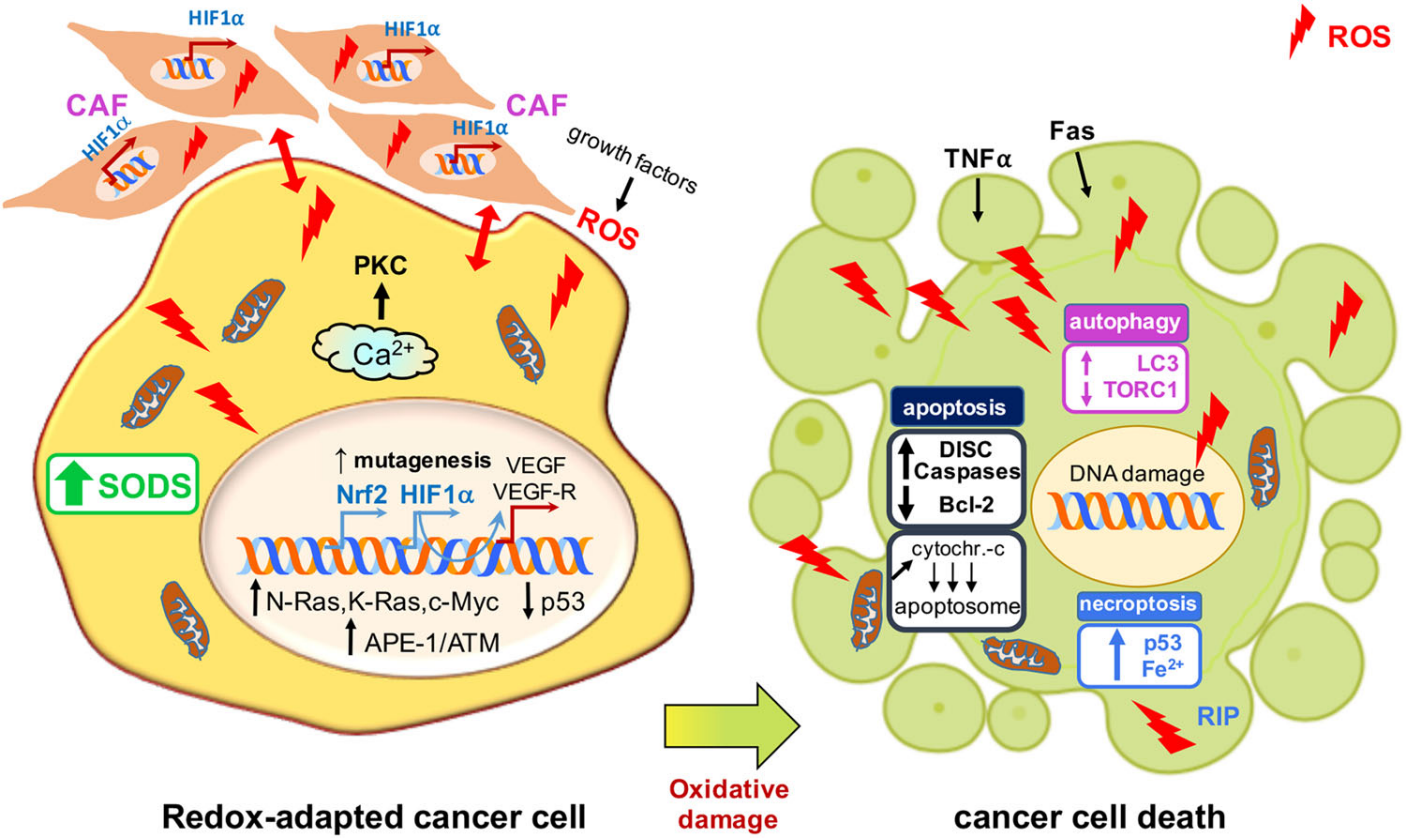
The three types of programmed cell death induced by elevated ROS levels in cancer cells. ROS, in response to death-inducing ligands (TNFα and Fas), enhance the assembly of DISCs and the activation of effector caspases and reduce Bcl-2 activity or, as a consequence of increased permeability of mitochondrial PTPs, stimulate the intracytoplasmic release of cytochrome c, which interacts with Apaf-1 and procaspases and forms the apoptosome (apoptosis). ROS can also inhibit the negative regulators of autophagy (TORC1) and increase the formation of LC3-dependent autophagosomes (autophagy). Finally, high levels of ROS, induced by several receptor-interacting protein kinases (RIPs), increase p53 expression, which increases ROS levels *via* a mechanism that depends on intracellular iron (ferroptosis).

Several noncancer cells associate with tumors: among these, cancer-associated fibroblasts (CAFs), particularly represented in the tumor microenvironment (TME), actively contribute to the regulation of tumor homeostasis, promoting tumor progression and the invasion of cancer cells. CAFs and ROS engage in two-way cross-talk: on the one hand, fibroblasts are targeted by ROS, particularly H_2_O_2_, which is able to convert them into active CAFs through the upregulation of HIF1α; on the other hand, CAFs are critical for the increase in ROS levels observed in cancer^50,51^. CAFs can also promote cancer growth and invasiveness, and both CAFS and ROS are linked through the increases in ROS-generated CAFs to which most cancers respond by increasing the expression of antioxidant genes^52–54^ (Fig. 2).

However, a growing body of evidence supports the view that antioxidant activities are essential for tumorigenesis. It has been recently reported that targets of the *Nrf2* gene, such as HMOX1, facilitate cancer development because they counteract the effect of oxidative stress in transformed cells^55^. Moreover, established oncogenes such as *K-RAS* and *c-MYC*, which had been previously demonstrated to induce intracellular ROS^56,57^, have been recently shown to stabilize Nrf2^58^. In this regard, mutations to *NRF2* and its regulator *KEAP1* have been found in cancer cells, supporting the supposition that antioxidant genes are pivotal in tumor progression^59–62^. In fact, it has been found that the breast cancer susceptibility 1 (*BRCA1*) gene interacts with and induces Nrf2 expression with positive outcomes on cancer cell survival^63^. Interestingly, estrogen stimulation of breast cancer cells that do not express BRCA1 and, as a result, suffer from high intracellular ROS levels rescues *NRF2* transcription, enhancing the survival of these cancer cells^64^.

Additionally, FOXO transcription factors have recently been implicated in tumorigenesis: in fact, rhabdomyosarcomas present FOXO genes with a high percentage of mutations that render them insensitive to inhibition by AKT signaling^65^. Moreover, increased intracellular levels of GSH are required for the initiation and progression of various types of cancer, and inhibitors of GR behave as anticancer drugs^66^, while high levels of NADPH boost the metastatic ability of melanoma cells, and protocols based on depletion of GSH (isothiocyanates and aziridine derivatives that bind GSH) or based on blocking the uptake of a rate-limiting precursor of its synthesis (inhibitors of the cysteine/glutamate antiporter, XCT) greatly impact cancer cell survival^67,68^. Specifically, sulfasalazine, an XCT inhibitor, appears useful in the treatment of pancreatic and small-cell lung cancer cells^69,70^, while NOV-002, a glutathione disulfide mimetic that alters the GSSG/GSH ratio and induces oxidative stress, has been favorably used in patients with HER2-negative breast cancer^71^. In addition, inhibitors of the enzyme glutaminase (GLS) that converts glutamine to glutamate, which is subsequently transformed to GSH *via* the glutamate–cysteine ligase complex, efficiently induce cancer cell death through dysregulation of their antioxidant system^72^. As mentioned above, another central player in these redox systems is thioredoxin, which is reduced by NADPH to induce the transfer of electrons for use in DNA synthesis, signal transduction and redox regulation. Interestingly, auranofin, which functions as a thioredoxin inhibitor, has been used with beneficial effects in the treatment of head and neck carcinoma cell lines; prevention of this effect by the ROS scavenger N-acetylcysteine (NAC) confirms the role of ROS in these cancers^73^.

### ROS and apoptosis (type I programmed cell death)

The most common method by which ROS kill transformed cells is the activation of PCD, which is completed within less than 60 min by a family of cysteine-dependent aspartate-directed proteases known as caspases. Triggered by an extrinsic or an intrinsic pathway, caspase-induced PCD culminates with the formation of apoptotic bodies that are eliminated by adjacent phagocytes^74^. The extrinsic pathway is mediated by binding of death-inducing ligands such as TNFα and Fas ligand that bind to cognate receptors that, in turn, recruit adaptor proteins and pro-caspases, leading to the assembly of the death-inducing signaling complex (DISC) and the activation of effector caspases^75^. This interaction is competed by the cellular FLICE-inhibitory protein (c-FLIP): ROS have been shown to downregulate the c-FLIP half-life by inducing its ubiquitin-proteasomal degradation, thus enhancing this extrinsic pathway^76^. However, compelling evidence suggests that, for the majority of ROS-related anticancer drugs, apoptosis depends on the activation of the intrinsic pathway that involves mitochondrial PTPs, the permeability of which is increased with the cytoplasmic release of pro-apoptotic factors such as cytochrome c that forms a complex with apoptotic protease activating factor 1 (Apaf-1) and pro-caspase 9 to build the apoptosome, activating, in turn, effector caspases^77–80^ (Fig. 2).

In fact, ROS induce the three major components critical for the opening of the PTPs, the voltage-dependent anion-selective channel (VDAC), adenine nucleotide translocase (ANT) and cyclophilin D, via the oxidation of specific cysteines in their active sites^81,82^. ROS also trigger apoptosis by inactivating or increasing the ubiquitination of the pivotal anti-apoptotic protein Bcl-2 and by decreasing the intracellular levels of Bax and Bad^83,84^ (Fig. 2).

The induction of apoptosis by elevated ROS levels has been highlighted as the central mechanism responsible for the positive effects of monoclonal antibodies^85^ and tyrosine kinase inhibitors^86^, which represent the core of targeted cancer therapy^87^. Among tyrosine kinase inhibitors, imatinib (a PDGFR inhibitor) and erlotinib (an EGFR inhibitor) induce ROS-dependent apoptosis in melanoma and non-small-cell lung cancer cells, respectively, through disruption of mitochondrial membrane potential upon the stimulation of JNK and p38 phosphorylation^88,89^, while vemurafenib (a BRAF inhibitor) increases the production of superoxide anions with the commensurate depolarization of the mitochondrial membranes in melanoma cells^90^. Among the monoclonal antibodies, rituximab (specific to the calcium-channel protein CD20 on the surface of B cells and mature plasma cells) increases ROS and induces apoptosis via the inhibition of Bcl-2 and p38MAPK signaling and is used in the treatment of B cell lymphomas^91^.

As noted above, chemotherapy and radiotherapy cause an increase in intracellular ROS that can lead to apoptosis^92,93^ *via* extrinsic or intrinsic pathways^94,95^. Many drugs used in anticancer therapy induce oxidative stress. Apoptosis is stimulated by procarbazine, which induces oxidative DNA damage that cannot be repaired by the BER/NER system in Hodgkin’s lymphoma and brain cancers^96^. Doxorubicin-dependent cytotoxicity is linked to the stimulation of a Fenton reaction that generates hydroxyl radicals successfully used for the treatment of Kaposi’s sarcoma, breast and bladder cancer and acute lymphocytic leukemia^97^. A course of treatment with arabinocytosine, which hampers DNA replication, followed by anthracyclines to increase ROS, has been shown to drive PCD, with beneficial effects for patients with acute myeloid leukemia (AML)^98^.

Arsenic trioxide has recently carved out a role in cancer therapy because it can induce electron leakage along the respiratory chain^99^. It triggers apoptosis in different cancer cells, including those of myeloma, lung cancer, and leukemia^100,101^. Moreover, 5-fluorouracil, a pyrimidine analog, produces ROS through p53-dependent pathways and induces apoptosis in colon and rectal cancer cells^102,103^.

ROS-induced apoptosis also explains the beneficial effect of two analogs of nuclear receptor ligands in several types of cancer: 2-methoxyestradiol, a 17β-estradiol metabolite, and N-(4-hydroxyphenyl) retinamide, a synthetic analog of retinoic acid, have been shown to induce PCD in neuroblastoma and lung cancer cells, respectively^104,105^. Furthermore, platinum-based drugs elevate ROS levels that promote PCD; protocols for the administration of these compounds in combination with inhibitors of poly(ADP-ribose) polymerase (PARP), which is involved in the maintenance of DNA integrity, have been shown to arrest the growth of breast cancer cells, even in *BRCA*-deficient models^106,107^. Intuitively, the inhibition of DNA damage repair by PARP may sensitize cancer cells to the oxidative stress induced by platinum-containing drugs.

Programmed cell death may also be mediated by the effect of elevated ROS on sphingomyelinase, which generates ceramide from sphingomyelin and binds to death receptors on the cell membrane of cancer cells. Activation of this pathway has been observed after UV irradiation of lymphoma cells^108^. Moreover, the use of drugs affecting mitochondria, where more than one-half of all ROS are generated, represents a suitable approach to induce oxidative stress and PCD in cancer cells^109^: gamitrinib, an inhibitor of heat shock protein 90 (HSP90), induces a dramatic collapse of mitochondria in prostate cancer cells^110^, while ARQ 501 (a quinone derivative) and STA-4783 (a copper chelator) increase ROS through leakage in the electron transport chain and have beneficial effects in patients with solid tumors and pancreatic adenocarcinoma^111^.

Apoptosis is triggered in cells with excessive endoplasmic reticulum (ER) stress that is induced when the protein folding ability of the ER is overwhelmed or impaired. Recently, several drugs have been designed on the basis of their ability to aggravate ER stress in cancer cells via the induction of oxidative stress. Among these, bortezomib is a proteasome inhibitor that induces ROS and ER stress in head and neck squamous cell carcinoma cells^112^, and celecoxib, a nonsteroidal anti-inflammatory drug, aggravates ER stress and induces apoptosis by altering the Bax/Bcl-2 ratio and increasing ROS in prostate cancer cells^113^.

### ROS and autophagy (type II programmed cell death)

Recently, an important therapeutic approach to kill cancer cells has been presented by ROS-induced autophagy^114^. Specifically, it has been reported that H_2_O_2_-dependent inactivation of autophagy-related gene-4 (*ATG4*) increases LC3-associated autophagosomes and that ATM-mediated oxidation of AMP-activated protein kinase (AMPK) inhibits mammalian target of rapamycin 1 (TORC1), a pivotal negative regulator of autophagy^115–117^ (Fig. 2). Indeed, autophagy, also known as type II programmed cell death, is now considered not only as a cell survival mechanism but also a tumor suppressor mechanism that induces the death of transformed cells^118^. In this regard, it has been reported that H_2_O_2_ induces autophagic cell death in glioma cells after treatment with the polycyclic ammonium ion sanguinarine, which increases electron leakage from mitochondria and induces NOXs^119^. Rapamycin, administered in combination with inhibitors of HSP90, causes mitochondrial damage with accompanying oxidative stress and autophagy and reduces tumor growth in *RAS*-dependent tumors^120^.

### ROS and necroptosis (type III programmed cell death)

ROS are also able to induce necrosis, which was originally considered an unregulated form of cell death but is now recognized as type III programmed cell death (necroptosis)^121,122^. ROS generated after the formation of ceramide or after an increase in energy metabolism induced by several receptor-interacting protein kinases (RIPs), either in the mitochondrial ETC and/or by NOXs, have been reported to enhance necroptosis^123–125^.

In addition, a very intriguing ROS-related molecular mechanism of tumor suppression by p53 has recently been highlighted; this protein induces a peculiar form of cell death, now called ferroptosis, via an increase in ROS levels that subsequently inhibit the cystine uptake typically mediated by the repression of a key component of the cystine/glutamate antiporter^126^. Ferroptosis depends on the presence of intracellular iron and is induced by ROS^127^ (Fig. 2). Therefore, the role of p53 in this context appears to be different from that reported in several studies showing that it decreases the levels of ROS. A plausible explanation of this apparent dichotomy is that p53 promotes cell survival by preventing excessive increases in ROS under moderate oxidative stress, whereas when the oxygen species increase over a threshold level, it switches to becoming a ROS inducer, triggering cell death. On the basis that ferroptosis is considered an oxidation-induced cell death mechanism, several trials with different drugs that elicit this pathway have been conducted^128,129^. Erastin is a synthetic drug that induces cell death through ferroptosis in tumor cells bearing mutant *RAS* by increasing intracellular ROS levels and altering the permeability of the outer mitochondrial membrane^130,131^.

### ROS and multidrug resistance

Increased ROS levels are thought to impair the multidrug resistance of cancer cells, which causes cancer development and metastasis during or after chemotherapy^132,133^. It has been recently shown that efflux pumps in the plasma membrane of cancer cells are crucial for the extracellular efflux of anticancer drugs^134^. These pumps belong to the adenosine triphosphate (ATP)-binding cassette (ABC) transporter superfamily and are dependent on intracellular ATP stores^135^. ATP is accumulated by a synthase driven by a proton gradient generated in mitochondria by the NADH-dependent electron transport chain^136,137^; therefore, one possible way to overcome efficient efflux in cancer drugs is to inhibit ATP synthesis by promoting NADH conversion to NAD through lipid membrane-coated silica carbon nanoparticles that, under near-infrared laser irradiation, target mitochondria and produce ROS with simultaneous consumption of NADH^138^.

### Nuclear ROS: a Trojan horse that induces DNA damage

**Fig. 3:**
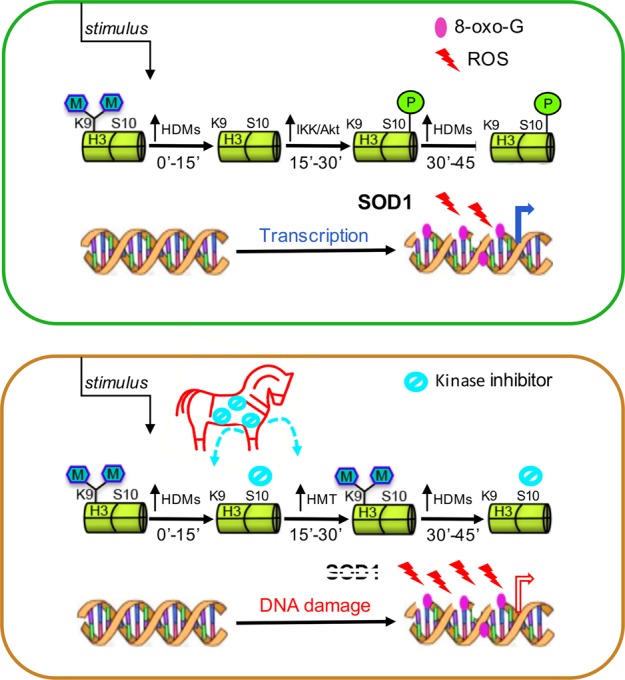
Role of nuclear ROS in transcription and DNA damage. ROS generated during nuclear receptor-induced transcription of target genes by the activity of lysine demethylases on lysine 9 in histone H3 must be controlled to prevent their accumulation. To this end, SOD1 reaches the nuclear space, while phosphorylation of H3S10 inhibits the rapid remethylation of the same lysine. If inhibitors of the H3S10 kinases are introduced as a Trojan horse together with nuclear receptor ligands, remethylation of H3K9 is quick, nuclear ROS accumulate, and unrepaired DNA damage triggers PCD.

A new role of ROS related to transcriptional output has been recently highlighted. It is well known that cells follow a strictly scheduled program for differentiation that is based on an orchestrated sequence of gene expression. Because of spatial constraints, genes must engage in a complex unfolding process to become accessible to the transcriptional machinery, which is triggered through posttranslational modifications at the N-terminal tails of core histones. Together, these modifications, induced by coordinated targeting of transcription factors that is currently referred to as epigenetic marks, conform to a precise code with specific time requirements to control whole gene expression^139^. We have previously shown that estrogen-induced transcription is triggered by LSD1-catalyzed demethylation of lysine 9 in histone H3 (H3K9), which is activated by the binding of liganded estrogen receptor to the enhancers of target genes^140^. This event is followed by the generation of ROS from the oxidation of FADH2 as induced by the demethylase, with consequent oxidation of nearby guanines (8-oxo-Gs) and recruitment of DNA repair enzymes (among which is APE1) that cause single-strand breaks in DNA and enable looping between the enhencer/promoter and the polyadenylation sites of the target genes, with productive transcription^140,141^. Intuitively, generation of ROS in this process must be timely and spatially controlled to prevent excessive damage to the DNA: a recent report, in fact, describes a new role for the originally discovered superoxide dismutase, SOD1, that is recruited to the nucleus in response to specific *stimuli*^142^. However, it has also been observed that hormone-induced phosphorylation of serine 10 in H3 histone (H3S10) prevents the rapid remethylation of the preceding lysine, serving as the metronome of the process and giving the DNA damage repair system enough time to eliminate the oxidized nucleotides from nearby DNA^143^. It has been reported that by inhibiting phosphorylation of serine 10 in this pathway, breast cancer cells simultaneously challenged with estradiol show an overproduction of ROS, with increased oxidation of the DNA that overwhelms the repair apparatus and triggers PCD in a great percentage of these cells^144^ (Fig. 3).

## Concluding remarks

**Fig. 4:**
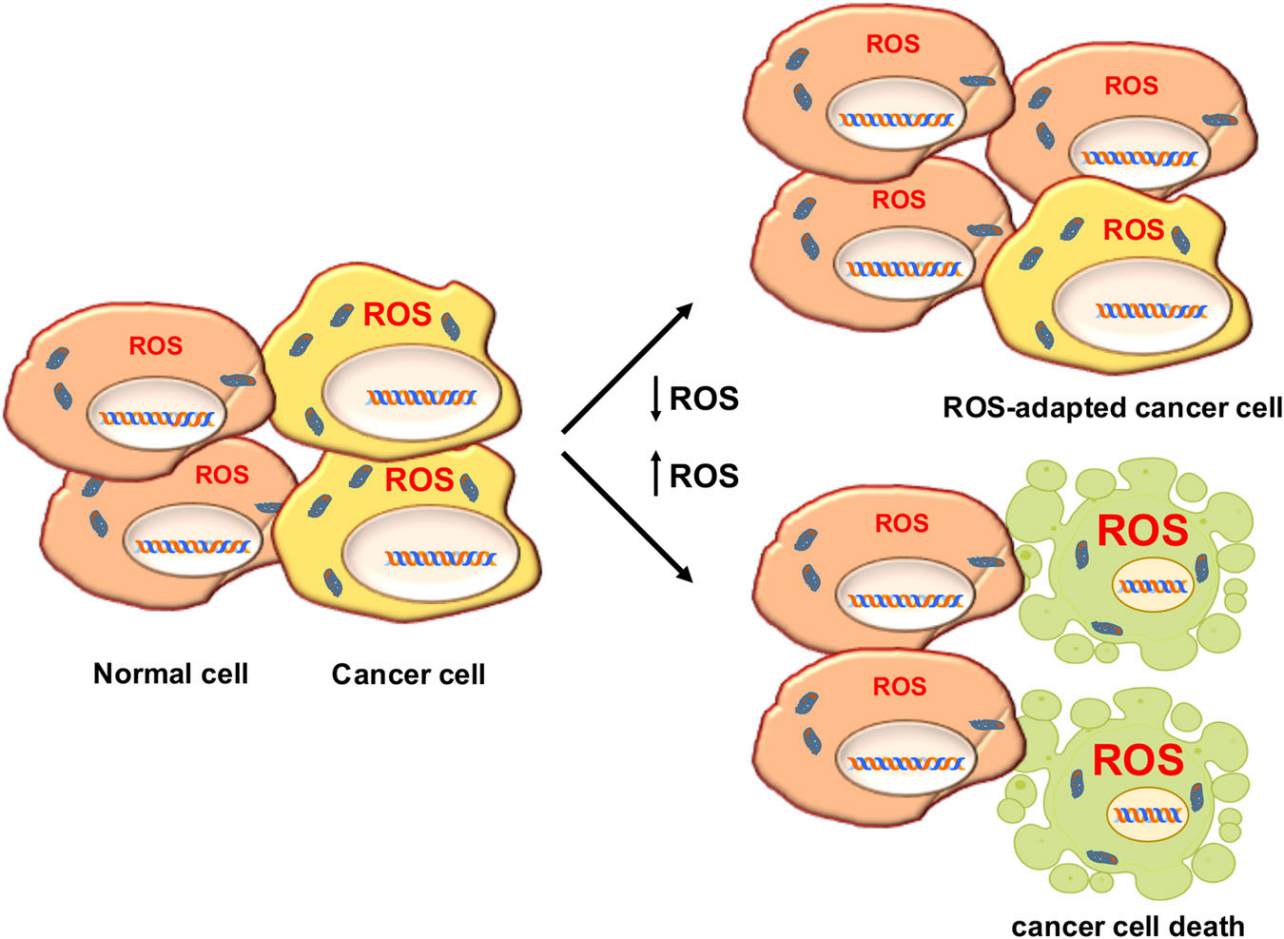
The two possible ROS-related anticancer therapeutic strategies. The first approach is based on lowering ROS levels to counteract their role in cellular transformation; it is aimed at reducing the number of transformed cells by depriving them of fuel (represented in the upper right side of the figure as a lower proportion of transformed cells with respect to that of normal cells). The second approach is based on the consideration that cancer cells, with an antioxidant system already triggered, are more sensitive than their normal counterparts to further increases in ROS and are unable to achieve redox balance. Therefore, by inducing ROS under these metabolic conditions, a high percentage of the cells undergo death (represented in the lower right side of the figure, where transformed cells are depicted as apoptotic).

The complex interconnection between ROS levels and cancer is essentially based on accurate fine-tuning between ROS production and scavenging. Cancer initiation and progression leverage slight increases in ROS levels. Therefore, cancer cells thrive on levels of ROS that are moderately higher than those in their normal counterparts, as they have developed increased antioxidant systems. This feature renders cancer cells more sensitive to external stimuli that further increase the production of ROS^145–147^, and, as schematically summarized in Table 1, an increasing number of therapeutic strategies are being developed to elevate ROS levels to overwhelm the redox adaptation of the same cells, inducing oxidative stress incompatible with cellular life^148–151^ (Fig. 4).

**Table 1:** List of anticancer drugs according to their effect on intracellular ROS, and types of cancer where are used.

Name	Mechanism to increase ROS	Cancers treated	Ref.
Sulfasalazine	XCT inhibitor	Pancreatic and small-cell lung cancer	^69,70^
NOV-002	GSSG mimetic	HER2-negative breast cancer	^71^
Imatinib (PDGFR inhibitor)	Loss of mitochondrial membrane potential	Melanoma	^88^
Erlotinib (EGFR inhibitor)	Loss of mitochondrial membrane potential	Non-small-cell lung cancer	^89^
Vemurafenib (BRAF inhibitor)	Depolarization of mitochondrial membrane	Melanoma	^90^
Rituximab (anti-CD20)	Inhibition of Bcl-2 *via* mitochondrial ROS	B-cell lymphoma	^91^
Procarbazine	Oxidized, generates ROS	Hodgkin’s lymphomas, brain cancer	^96^
Doxorubicin	Fenton’s reaction and electron leakage	Kaposi’s sarc, breast and bladder cancer, ALL	^97^
Arsenic trioxide	Electron leakage	Myeloma, lung cancer and leukemia	^100,101^
5-fluorouracil	P53-dependent ROS	Colon and rectal cancer	^102,103^
2-methoxyestradiol	Loss of mitochondrial membrane potential	Neuroblastoma	^104^
N-(4-hydroxyphenyl retinamide	Mitochondrial damage	Lung cancer	^105^
Platinum drugs	ROS-dependent DNA damage	Breast cancer (in ccombination with PARP inhibitors)	^106,107^
Gamitrinib	Mitochondrial collapse	Prostate cancer	^110^
ARQ 501 and STA-4783	Leakage of electron transport	Pancreatic adenocarcinoma and solid tumors	^111^
Bortezomib	ROS due to ER stress	Head and neck squamous cell carcinoma	^112^
Celecoxib	ROS after ER stress	Prostate cancer	^113^
Sanguinarine	Electron leakage and induction of NOXs	Glioma	^119^
Rapamycin	ROS from ER stress	*RAS*-driven tumors	^120^

Specifically, cellular responses to ROS must be imagined as the integration of multiple levels in which, in addition to their nature and relative concentration, their location plays an important role. In fact, mitochondrial ROS have been reported to essentially promote cell death, while NOX-generated ROS have been associated with the promotion of cell proliferation and migration^152^. Furthermore, in contrast to the mechanism of sister pathways, redox signaling is based on migrating electrons, and therefore, the signaling in this pathway is much more diffuse.

In reference to the nature of ROS behavior as a double-edged sword, even though several studies have documented the benefits of antioxidant drugs for cancer therapies, none has been supported by solid trials performed on a large scale^153,154^. In contrast, the most recent studies have shown an increase in tumor development and metastasis in mouse models treated with vitamin E^155^ (an opposite result of that in which high doses of vitamin C increase ROS levels to induce the death of colon cancer cells bearing *KRAS* and *BRAF* mutations)^156^. In addition, it has been shown that the administration of antioxidants, such as N-acetylcysteine, accelerates the progression of lung cancers and melanomas^146^ and that increasing the expression of the antioxidant-encoding *Nrf2* gene enhances the growth of lung tumors^157–160^.

In fact, and in contrast to the previous view, the results of many studies support a scenario in which the inhibition of antioxidant enzymes ensures the death of cancer cells, especially when this approach is used in combination with treatments that increase ROS. This approach is an alternative to the traditional strategy of targeting oncogenes and tumor suppressor genes, a strategy that appears complicated because of the high number of genes involved and their ability to drive compensatory pathways^161^.

Interestingly, increased ROS-induced apoptosis has been reported in cancer cells after depletion of ATP derived from the manipulation of glycolytic enzymes, chemotherapy or radiation therapy; these data highlight the potential eminent role of ROS modulation in anticancer combinatorial therapies^162,163^.

Finally, the most recent ROS-inducing drugs have addressed the pivotal goal of therapists: cancer selectivity. In this regard, good results have been reached through photodynamic therapy, which is based on the generation of ROS after stimulation of a photosensitizer by light: cancer cells under treatment internalize porphyrin precursor molecules to induce the formation of ROS that lead to photooxidative stress and cancer-specific cell death^164,165^. In fact, although more studies are required to increase the selectivity of these anticancer ROS-related drugs, the common mechanisms elicited by oncogenes to promote the adaptation to a large set of stress conditions are being revealed in more depth every day, and in a high percentage, they concern the redox balance.

In conclusion, we expect that targeting ROS will represent fruitful ground for future molecular anticancer strategies.
